# It's Not Over Till It's Over: A Prospective Cohort Study and Analysis of "Anesthesia Stat!" Emergency Calls in the Pediatric Post-Anesthesia Care Unit (PACU)

**DOI:** 10.7759/cureus.17571

**Published:** 2021-08-30

**Authors:** Susan R Vishneski, Moeko Nagatsuka, L D Smith, T W Templeton, Martina G Downard, Eduardo J Goenaga-Diaz, Leah B Templeton

**Affiliations:** 1 Department of Anesthesiology, Wake Forest School of Medicine, Winston-Salem, USA

**Keywords:** pediatric anesthesiology, patient safety, anesthesia, operating rooms, child, academic medical centers, emergencies

## Abstract

Background

Emergency "Anesthesia Stat!" (AS!) calls remain a common practice in medical centers even when advanced communication infrastructures are available. We hypothesize that the analysis of post-procedure "AS!" calls will lead to actionable insights which may enhance patient safety.

Methods

After institutional review board approval, we prospectively collected data from April 2015 through May 2018 on "AS!" calls throughout the pediatric operating rooms (OR), off-site locations, and post-anesthesia care unit (PACU) at a tertiary university medical center. Data recorded included demographic information, location, time of the event, event duration, vital signs, medications, anesthesia staff, attending anesthesiologist, and staff responding to the call. A narrative account of the event was also documented.

Results

A total of 82 "AS!" calls occurred, with ages ranging from 11 days old to 17 years old. Forty-nine of the 82 calls (60%) occurred at emergence. Seventy-one of the 82 calls (87%) were solely respiratory-related. Thirty-five of 49 emergence calls (71%) occurred in the PACU. Further, 34 of 35 PACU calls (97%) were respiratory-related, with 30 of 35 PACU calls (86%) associated with desaturation requiring intervention by anesthesia staff. Finally, 31 of 35 PACU calls (89%) occurred within 30 minutes of patient arrival to PACU.

Conclusion

Analysis of "AS!" events from our PACU continues to support the need for the prompt and continuous availability of at least one staff member with advanced airway management skills. Further, pediatric patients undergoing general anesthesia and surgery should likely be monitored for a minimum of 30 minutes following arrival in the PACU.

## Introduction

Overhead emergency calls remain a common practice at many institutions [1-3]. Even with the advent of cellphones and other two-way communication platforms, many institutions maintain an audible alert system to rapidly mobilize anesthesia resources to assist in the management of various critical events such as cardiac arrest or unanticipated difficult airway management. At our institution, "Anesthesia Stat!" (AS!), the primary overhead emergency call in the operating room (OR), post-anesthesia care unit (PACU), and off-site locations, has been in use for over three decades. Previous literature looking at emergency overhead calls has generally evaluated these events in an attempt to identify risk factors and stratify patients at risk for critical events [1-2].

In general, events requiring an overhead emergency call are rare. However, studying rare critical events may allow for improvements in patient care [4-5]. When compared to adult patients, pediatric patients may deteriorate faster in response to anesthesia-related adverse events, and therefore studying critical incidents is necessary to improve patient outcomes and safety [6-7]. Additionally, events requiring an overhead emergency call have been reported at every point from induction through emergence, including in areas where personnel with advanced airway management skills may not be immediately available [2,4]. As such, evaluating events that occur in the PACU may have the greatest impact on patient care.

Therefore, the primary aim of this study was to develop a descriptive analysis of AS! events, specifically those occurring during emergence and in the PACU, to garner insights that may lead to improvements in the post-procedure care of pediatric patients at our medical center. We further hypothesize that an analysis of the cause, timing, and resolution of these events will be informative for perioperative decision-making. For example, determining a safe minimum duration of post-procedure observation in the PACU.

This article was previously presented as a poster at SPA-AAP Pediatric Anesthesiology 2019 on March 17, 2019.

## Materials and methods

Following institutional review board approval, we prospectively observed AS! events in the pediatric ORs, PACU, and various off-site locations at Wake Forest Baptist Health, a tertiary medical center where all pediatric surgical disciplines are represented. In most cases, a dedicated member of the research team not immediately involved in patient care would observe the AS! event alerted by the emergency overhead call.

Events qualifying for inclusion were any event in the pediatric OR, off-site, or PACU for which an overhead AS! call in a child less than 18 years of age was made. Off-site locations include interventional radiology, computed tomography (CT), and magnetic resonance imaging (MRI). Events in patients greater than or equal to 18 years of age were not included in the analysis.

Data recorded for each event included age, weight, American Society of Anesthesiologist (ASA) classification, history of recent upper respiratory infection (URI), any change in vital signs, lowest oxygen saturation (SpO2), anesthesia staff (attending anesthesiologist, resident, or certified nurse anesthetist [CRNA]), and airway at time of AS! call (mask, supraglottic airway [SGA], or endotracheal tube [ETT]). Time of AS! call, anesthesia start and stop times, and duration of the event were also recorded. Additionally, we recorded the patient’s phase of care at the time when the call occurred (induction, maintenance, or emergence). We also recorded the location of the event, including whether it occurred in the OR, off-site, or PACU. The number and type of staff responding to the event were also recorded. Finally, a narrative describing the initial cause, interventions performed, and the final resolution was documented.

Data were collected during normal business hours (7 am to 5 pm, Monday through Friday). Events occurring at other times could be self-reported, but there was not a system in place to capture these events due to a lack of available study staff at these times.

Statistical analysis and sample size

Descriptive statistics, including medians/ranges for demographic data not normally distributed and frequencies and proportions for categorical data, were calculated. All comparisons of non-parametric data were done using the Mann-Whitney U-test for comparison of non-parametric data. P<0.05 was considered statistically significant. All available cases meeting inclusion criteria were used for analysis.

## Results

Following IRB approval, 82 AS! events were recorded between April 2015 and May 2018 in our pediatric ORs, off-site locations, and PACU. Demographic data and general event characteristics are presented in Table 1. The majority of calls, 71/82 (87%), were solely respiratory in nature, with 53/71 (75%) of these calls being associated with attendant desaturation below 90%. Out of the total of 82 AS! calls, four (4.9%) occurred during induction of anesthesia while 29 (35%) occurred during the maintenance phase of care. The majority of AS! calls, 49/82 (60%), occurred during emergence.

**Table 1:** Demographics and general event characteristics for all “Anesthesia Stat!” calls ASA = American Society of Anesthesiologists; URI = upper respiratory infection

	N = 82
Age (yr)	2 (11 days - 17 years)
<1	19 (23%)
>1	63 (77%)
Weight (kg)	13.6 (3.4 - 90.7)
	N (%)
ASA classification	
I	28 (34)
II	30 (37)
III	20 (24)
IV	4 (5)
History of asthma	10 (12)
Upper respiratory infection or symptoms of URI	17 (21)
Location of call	
Operating room	46 (56)
Post-anesthesia care unit	35 (43)
Off-site	1 (1)
Phase of anesthetic	
Induction	4 (5)
Maintenance	29 (35)
Emergence	49 (60)
Length of event (min)	4 (< 1 - 73)
Number of responders	3 (1 - 6)
Nature of call	
Respiratory only	71 (87)
Cardiovascular only	4 (5)
Combined respiratory and cardiovascular etiology	5 (6)
Other (emesis, seizure-like activity)	2 (2)

**Figure 1:**
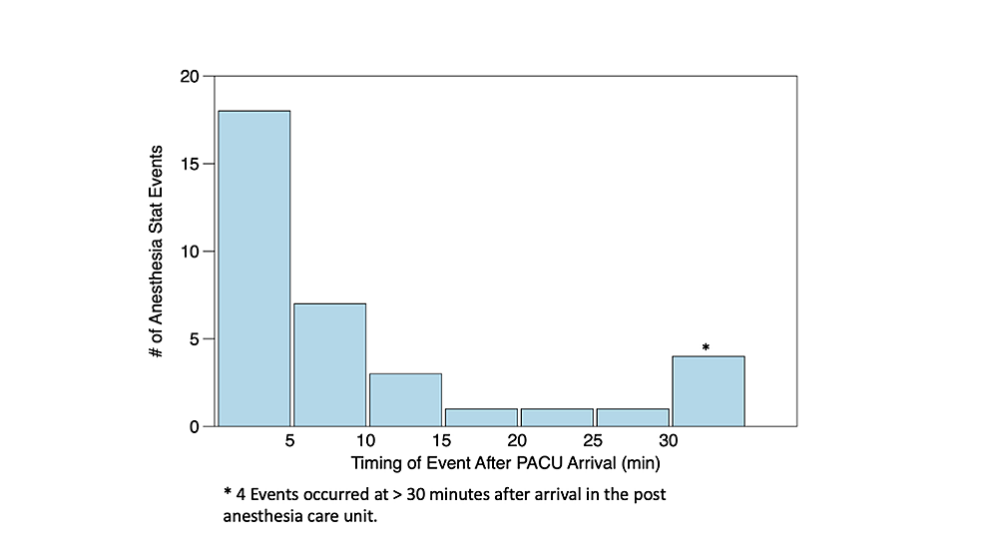
Timing of "Anesthesia Stat!" events in the post-anesthesia care unit *Four events occurred at greater than 30 minutes after arrival in the post-anesthesia care unit.

Of the emergence calls, 14/49 (29%) occurred while the patient was still in the OR while 35/49 (71%) occurred while the patient was in the PACU, and 20/35 (57%) of the PACU calls occurred after care had been transferred to a PACU nurse. A summary of event characteristics for the 35 PACU AS! calls can be found in Table 2. Almost all PACU AS! calls (31/35 [89%]) occurred within 30 minutes of PACU arrival, as presented in Figure 1. A total of two PACU calls were initiated greater than 60 minutes after admission to the PACU. Both of these calls occurred following the administration of opioid medications to these patients while they were awaiting an inpatient bed.

**Table 2:** Summary of characteristics associated with “Anesthesia Stat!” calls in the post-anesthesia care unit a. AS! calls occurring while the anesthesia team taking care of the patient in the operating room was still present in the PACU were considered to have started upon arrival in the PACU or at time 0 and not from the Anesthesia Stop time to avoid negative times. b. AS! calls in the PACU at greater than 60 minutes following the administration of opioid medication in the PACU in patients that were awaiting an inpatient bed. AS! = Anesthesia Stat!; URI = upper respiratory infection; PACU = post-anesthesia care unit

	N (%)
Age	
< 1 year old	2 (6)
> 1 year old	33 (94)
URI	
Present	7 (20)
Absent	26 (74)
Unknown	2 (6)
Airway present for procedure	
Endotracheal tube	16 (46)
Laryngeal mask airway	15 (43)
Mask	1 (3)
Unknown	3 (8)
Airway present at time of call	
Endotracheal tube	1 (3)
Laryngeal mask airway	0 (0)
Mask	34 (97)
Length of AS! call event (min)	5 (1 - 73)
Time elapsed from anesthesia stop to AS! call (min)	11 (0^a^ – 280^b^)
Called before anesthesia stop	15 (43)
Called after anesthesia stop	20 (57)

Interestingly, in children less than one year of age, 3/5 (60%) of the emergence-related AS! calls occurred in the OR. This is in contrast with older children where 33/44 (75%) of the emergence-related AS! calls occurred in the PACU. Overall, the median length of AS! events in the PACU in children less than one year of age was 8.5 (7-10) versus four (1-73) minutes in children greater than or equal to one year of age. When comparing the median number of staff who responded to a given AS! call, there was not a significant difference between the OR, 3 (1-6), and PACU, 4 (2-6) (P=0.23).

Looking at narrative comments documented at emergence, most calls in the OR tended to originate immediately following extubation, or very shortly thereafter, and were frequently associated with laryngospasm, apnea, or breath-holding. Of the 14 emergence calls that occurred in the OR, a total of eight (57%) received a pharmacologic intervention, specifically propofol 6/14 (43%), succinylcholine 4/14 (29%), atropine 2/14 (14%), epinephrine 2/14 (14%), and albuterol 1/14 (7%). Of the 35 emergence calls that occurred in the PACU, a total of 20 (57%) were managed with pharmacologic intervention in addition to different airway maneuvers such as jaw thrust, placement of an oropharyngeal airway, and/or continuous positive airway pressure (CPAP). Finally, 43% (15/35) of AS! calls in the PACU were in patients whose airways had been managed with an SGA during the procedure, which had been removed while the patient remained in a deep plane of anesthesia.

Twenty-one percent (17/82) of patients involved in an AS! event were reported to have URI or URI symptoms. The presence of URI did not appear to prolong the duration of AS! events with a median duration of five (1-6) minutes vs. four (1-73) minutes in children who did and did not have a URI, respectively (P=0.15).

A total of 6/82 (7%) events were considered non-respiratory in nature while an additional five events were considered to have combined cardiovascular and respiratory etiologies. Overall, these events included episodes of bradycardia, cardiac arrest, significant hypotension, vomiting, shivering resembling seizure-like activity, and loss of intravenous access. A majority, 6/11 (55%), of these events occurred at induction or during the maintenance phase of care while 5/11 (45%) occurred during emergence. Of these, 3/11 (27%) received cardiopulmonary resuscitation with a return of spontaneous circulation.

## Discussion

The primary finding of this study was that nearly half of AS! calls originated in the PACU during the period of observation. Further, many of these calls took place after a hand-off from the anesthesia staff to the PACU nurse, and the vast majority were directly related to respiratory embarrassment. These events, such as breath-holding, laryngospasm, and upper airway obstruction, frequently led to desaturation and required a variety of different interventions from CPAP and bag-mask ventilation to the administration of propofol and/or succinylcholine. Thus, it remains essential that personnel with advanced airway management skills be available to assist in these settings. This should be taken into account as facilities contemplate safe and efficient staffing models. Given the frequency of these events, it is important to ensure the ability to address very treatable respiratory issues so that they do not lead to more critical events. One solution may be a care team approach to ensure that at least one person with advanced airway management skills is available to respond to these events [2,8].

Another interesting finding in our study was that the vast majority of calls related to the emergence phase following general anesthesia occurred within 30 minutes of arrival to the PACU. This was inclusive of all patients, even those in whom an SGA was removed while the patient remained in a deep plane of anesthesia. This is significant, as there remains no consensus within the pediatric anesthesia community in terms of precisely how long a given patient should be monitored following general anesthesia and surgery. This is especially relevant in outpatient settings where increased efficiencies must be balanced against issues of patient safety. For example, in the setting of some otolaryngologic procedures, such as tonsillectomies, patients may be at a higher risk of adverse respiratory events in the PACU [9]. Given that most PACU events appear to occur within 30 minutes, it would appear a reasonable guideline to monitor pediatric patients for a minimum of 30 minutes following general anesthesia.

Interestingly, about half of the patients with an AS! call occurring in the PACU had been managed intraoperatively with an SGA and the other half had been managed intraoperatively with an ETT. This was despite the fact that all ETTs were removed while the patient was awake in the OR while all SGAs were removed while the patient was still in a deep plane of anesthesia. One might naturally hypothesize that patients in whom an SGA was removed in a deeper plane of anesthesia would be at increased risk of a respiratory event post-procedure, as they may remain in stage 2 of anesthesia without a protected airway for a significant period of time while in the PACU. Overall though, it is difficult to make firm statements on the risk of using an SGA versus an ETT with these data. The overall usage of both devices was not recorded as part of the study. Therefore, it is difficult to make any statement on the incidence or prevalence of these events with a given device.

Previous studies looking at emergency events in anesthesia have highlighted a variety of issues. For example, one of the most common findings is that a majority of emergency events appear to originate from respiratory events [10-13]. Other studies of emergency events in anesthesia have focused on interventions and educational programs in an attempt to improve patient outcomes. One study by Vlassakova et al. examined 193 emergency calls initiated by perioperative personnel over a four-year period and found that emergency calls initiated by an attending or fellow were much more likely to be complicated events, as compared to those initiated by anesthesia residents, CRNAs, or nursing staff [2]. They hypothesized that this finding is most likely due to the different degrees of experience acting as a driving force to call for additional help. In response to their study, premature infants with complicated medical histories are now assigned to more experienced anesthesia staff, such as fellows and upper-level residents, at that center [2]. Another study conducted in India analyzed 108 pediatric anesthesia critical events over a one-year period and found that 63% of their airway-related events occurred at recovery [14]. The institution of this study went on to develop a variety of safety practice guidelines that included color-coding drugs, enforcing early calls for help in difficult airway situations, and ensuring the visibility of IV lines.

Limitations

The limitations of our study include the fairly small sample size and the fact that it represents a single center’s experience. Additionally, reporting of events was subject to the availability of study staff and therefore was unlikely to capture off-hour events unless they were self-reported by the anesthesia staff. In most cases though, events were recorded either in real-time or shortly after the event. Additionally, because we did not record the overall utilization of individual airway management approaches, it is difficult to know if the use of an SGA or ETT was actually more or less predisposed to post-procedure events, given that one device may have been used with much greater frequency than the other. Importantly though, it would appear that both approaches can lead to post-procedure respiratory complications.

## Conclusions

In summary, post-procedure AS! calls appear to represent a significant proportion of overhead emergency calls in our ORs and off-site locations. Further, an analysis of these calls is instructive in developing strategies to improve patient safety such as maintaining the routine availability of a staff member with advanced airway management skills who is not simultaneously engaged with the care of a patient in an OR. Additionally, these results suggest that institutions should consider mandating a minimal period of post-procedure observation that is likely to include the vast majority of critical post-procedure events. Finally, centers should continue to monitor critical events like overhead emergency calls to gain insight into rare but significant events, which may lead to process or system-level changes that may ultimately improve patient care and patient safety.
